# Integrating Mobile Phones into Medical Abortion Provision: Intervention Development, Use, and Lessons Learned From a Randomized Controlled Trial

**DOI:** 10.2196/mhealth.3165

**Published:** 2014-02-14

**Authors:** Katherine Marianne de Tolly, Deborah Constant

**Affiliations:** ^1^Cell-LifeCape TownSouth Africa; ^2^Women’s Health Research UnitSchool of Public Health and Family MedicineUniversity of Cape TownCape TownSouth Africa

**Keywords:** mHealth, telemedicine, SMS, medical abortion, USSD, mobisite

## Abstract

**Background:**

Medical abortion is legal in South Africa but access and acceptability are hampered by the current protocol requiring a follow-up visit to assess abortion completion.

**Objective:**

To assess the feasibility and efficacy of information and follow-up provided via mobile phone after medical abortion in a randomized controlled trial (RCT).

**Methods:**

Mobile phones were used in three ways in the study: (1) coaching women through medical abortion using short message service (SMS; text messages); (2) a questionnaire to assess abortion completion via unstructured supplementary service data (USSD, a protocol used by GSM mobile telephones that allows the user to interact with a server via text-based menus) and the South African mobile instant message and social networking application Mxit; and (3) family planning information via SMS, mobisite and Mxit. A needs and context assessment was done to learn about women’s experiences undergoing medical abortion and their use of mobile phones. After development, the mobile interventions were piloted. Recruitment was done by field workers at the clinics. In the RCT, women were interviewed at baseline and exit. Computer logs were also analyzed. All study participants received standard of care at the clinics.

**Results:**

In the RCT, 234 women were randomized to the intervention group. Eight did not receive the intervention due to invalid numbers, mis-registration, system failure, or opt-out, leaving 226 participants receiving the full intervention. Of the 226, 190 returned and were interviewed at their clinic follow-up visit. The SMSs were highly acceptable, with 97.9% (186/190) saying that the SMSs helped them through the medical abortion. In terms of mobile phone privacy, 86.3% (202/234) said that it was not likely or possible that someone would see SMSs on their phone, although at exit, 20% (38/190) indicated that they had worried about phone privacy. Having been given training at baseline and subsequently asked via SMS to complete the self-assessment questionnaire, 90.3% (204/226) attempted it, and of those, 86.3% (176/204) reached an endpoint of the questionnaire. For the family planning information, a preference for SMS was indicated by study clients, although the publicly available Mxit/mobisite was heavily used (813,375 pages were viewed) over the study duration.

**Conclusions:**

SMS provided a good medium for timed, "push" information that guided and supported women through medical abortion. Women were able to perform a self-assessment questionnaire via mobile phones if provided training and prompted by SMS. Phone privacy needs to be protected in similar settings. This study may contribute to the successful expansion of medical abortion provision aided by mobile phones.

**Trial Registration:**

Pan African Clinical Trials Registry (PACTR): PACTR201302000427144; http://www.pactr.org/ATMWeb/appmanager/atm/atmregistry?dar=true&tNo=PACTR201302000427144 (Archived by WebCite at http://www.webcitation.org/6N0fnZfzm).

## Introduction

### Background Information

Medical abortion is legal in South Africa, and services have recently been expanding in the public sector. Medical abortion has support from both clients and providers [1-4]. Reasons given by clients included privacy, control of the process by the client, and avoidance of surgical procedures. Providers of surgical abortion in the public sector felt that medical abortion could help relieve the burden on current services [3] because medical abortion is more acceptable to nursing staff with ambivalent feelings about abortion.

The current protocol requires a follow-up appointment to assess abortion completion, and in practice the regimen of clinic visits required for medical abortion is higher than that for surgical abortion [2,5], and substantial staff time can be spent tracing clients who have defaulted on their follow-up appointments ([6]; personal communication by Deborah Constant with medical abortion provider, 2010). From a client perspective, each additional clinic visit can be a burden to those who have difficulty accessing abortion services due to time and/or economic constraints (eg, working women, women at school, or women living far from clinics). Previous research has shown that this visit can be replaced with a telephone assessment [7-9].

### Mobile Phones in South Africa

South Africa has a very high level of mobile phone ownership. International Telecommunications Union (ITU) [10] statistics indicated that there were 134.8 pre- and post-paid mobile subscriptions for every 100 people in 2012. South Africa’s All Media and Products Survey (AMPS) reported in 2011 that 80% of adults personally own, rent, or use a mobile phone, while only 15% have a landline telephone at home [11]. In terms of gender, there is only a 5% difference in favor of men in terms of mobile ownership [12].

There is little published research for South Africa on shared versus private use of mobile phones. One study of 411 low-income urban youth in Cape Town found that more than three-fourths (77%) of respondents reported that they owned a personal handset rather than using or sharing someone else’s phone (18%) [13]. A small minority used someone else’s phone but owned a personal SIM card (4%). The ITU’s and AMPS’ high rates of mobile penetration in South Africa tally with the findings of Kreutzer’s study: the majority of South Africans have their own mobile phone.

### Use of Mobile Phones in Health

mHealth literature can provide evidence for efficacy from similar interventions and suggestions for good practice. Particularly relevant is a review of research evaluations of mobile phone voice and text message interventions that provided care and disease management support, which looked at outcomes of care and processes of care [14]. The authors reported, “[s]ignificant improvements were noted in compliance with medicine taking, asthma symptoms, HbA1C, stress levels, smoking quit rates, and self-efficacy. Process improvements were reported in lower failed appointments, quicker diagnosis and treatment, and improved teaching and training.” Importantly, they stated that enhancing current standards of care with reminders, disease monitoring and management, and information provision through mobile phones can help improve health outcomes and care processes, and that this has implications for both patients and providers.

The recent review of mHealth behavior change communications interventions in developing countries [15] emphasized the importance of formative research to design interventions that are appropriate for the audience from a cultural perspective and in terms of health needs and telephone use. The review also recommended pretesting of messages to test these elements.

While the number of studies on the use of mobile phones in health has grown rapidly in the past few years, there are significant gaps in the mHealth body of evidence [16], and investigation into the use of telephones in medical abortion was limited to the use of voice calls in verbal consultative follow-up [7,8]. To our knowledge, no studies into the use of text-based mobile phone interventions for medical abortion follow-up were known at the time of designing this study.

### Purpose

To explore the use of mobile phones to simplify medical abortion provision, a study was done in South Africa to assess the feasibility and efficacy of a package of information, self-assessment of abortion completion, and support provided via mobile phones to reduce the need for a follow-up visit by clients, enhance the experience of medical abortion for clients, reduce demands on medical abortion providers; and strengthen post-abortion knowledge and uptake of family planning.

This paper describes the process of developing the various mobile interventions used in the randomized controlled trial (RCT), reports on findings of the study related to mobile phone use in this context, and reflects on lessons learned. It is anticipated that this will contribute to the growing body of knowledge around the use of mobile phones in health, and in particular the use of mobile phones to make a medical procedure easier.

## Methods

### Study Phases

The study involved three phases: a needs and context assessment (NCA), a pilot, and an RCT (PACTR201302000427144). Development of the mobile interventions was done after the NCA.

### Needs and Context Assessment

The NCA was designed to learn about the experiences of South African women undergoing medical abortion to inform the content of text messaging (short message service, SMS). We also wanted to understand women’s use of their mobile phones to design the mobile interventions suitably. Twenty consenting clients were asked at their medical abortion follow-up appointment about their experience of their medical abortion, current use of their mobile phones, the privacy of their mobile phone, their language preference for texting, and their feedback on draft text messages was solicited. Feedback on the draft text messages was sought by sending participants one text message, and showing them other text messages in mobile phone mock-ups.

Six medical abortion providers were also interviewed to learn more about issues they faced in medical abortion provision and their impressions of challenges faced by clients, so that these could be addressed in the mobile interventions. The NCA was done in May and June 2011 at a nongovernmental clinic in Cape Town’s city center and a public sector clinic in Khayelitsha, a disadvantaged township area 20 km outside the city.

### Development of the Mobile Interventions

#### Rationale

The development of the mobile interventions used the information gleaned from the NCA. Mobile was used in three ways in the study: (1) text messages coaching women through the medical abortion process; (2) self-assessment of abortion completion; and (3) provision of family planning information and encouragement via a mobisite (website optimized for mobile phones), Mxit (a popular South African mobile instant message chat and social network with over 7.5 million active users) [17], and text messaging.

These mobile-based interventions were all text-based and automated so that no additional burden was placed on health care providers, and the interventions could be easily scaled if effective or useful.

#### Text Messaging

Text messages were chosen for coaching because they can be timed (ie, sent at a particular time and date), and are a private means of communication if recipients have their own phones (which is largely the case in South Africa).

The text messages were sent via Cell-Life’s online system Communicate, which allows for text messaging campaigns to be preloaded on the system and triggered by registering a new study participant on the system. (Hence, text messages did not need to be sent manually for each participant; she just needed to be subscribed to the relevant Communicate SMS campaign, and her text messages were automatically sent by the system.) Text messages were set to be sent at particular times (at 9 am and 6 pm), and participants had been told what time to expect the text messages in a welcome-SMS.

**Figure 1:**
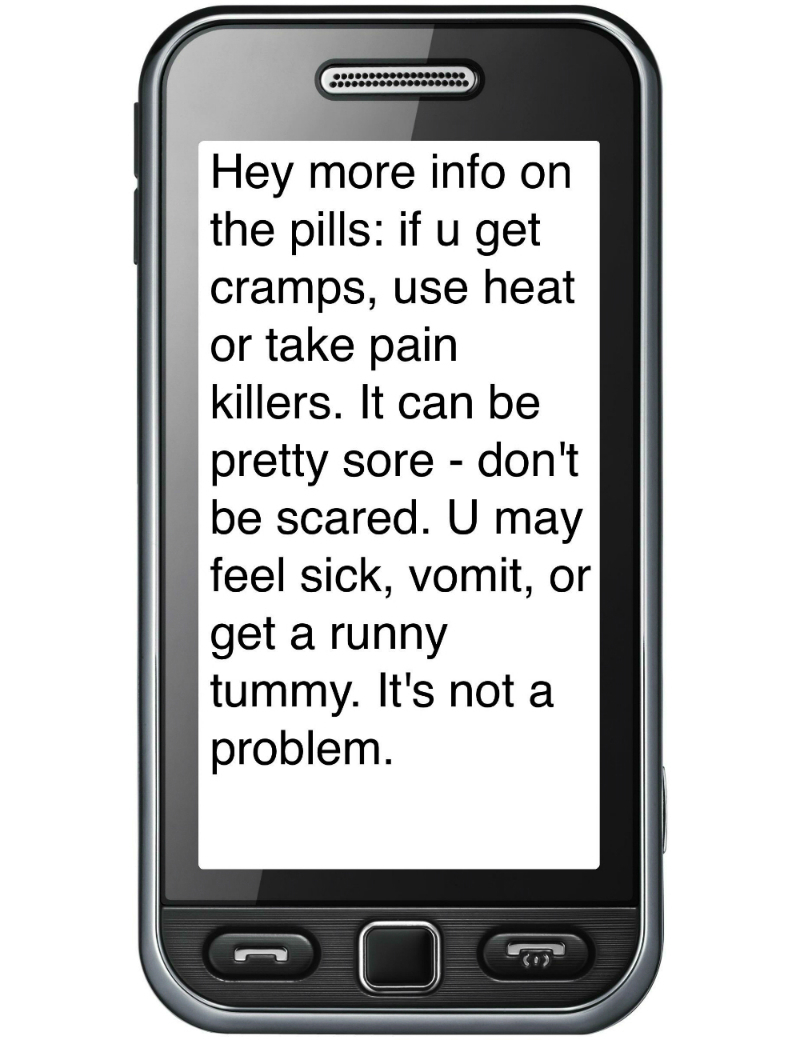
Example of text message.

SMS text messaging content was developed based on a literature review that covered how women experience the medical abortion process (eg, timing of bleeding and pain, and potential danger signs like excessive bleeding and fever), and issues raised by women and providers in the NCA (Figure 1). The text messages were provided in English only in the pilot, and an additional two South African languages in the RCT (Xhosa and Afrikaans).

In the pilot and RCT, each participant received 23 text messages over approximately 3 weeks. Nineteen of these were double SMSs; that is, two concatenated into one to allow for a longer text message.

Participants could opt out of receiving text messages at any point by sending a free please-call-me text message to a number that had been provided in the first text message they received after consenting to the study. This is standard procedure in South Africa—when people subscribe to a text messaging service, they should be provided with a way to stop the text messages. This was particularly important in our study, in case women had privacy concerns arising from the SMSs.

#### Self-Assessment

**Figure 2:**
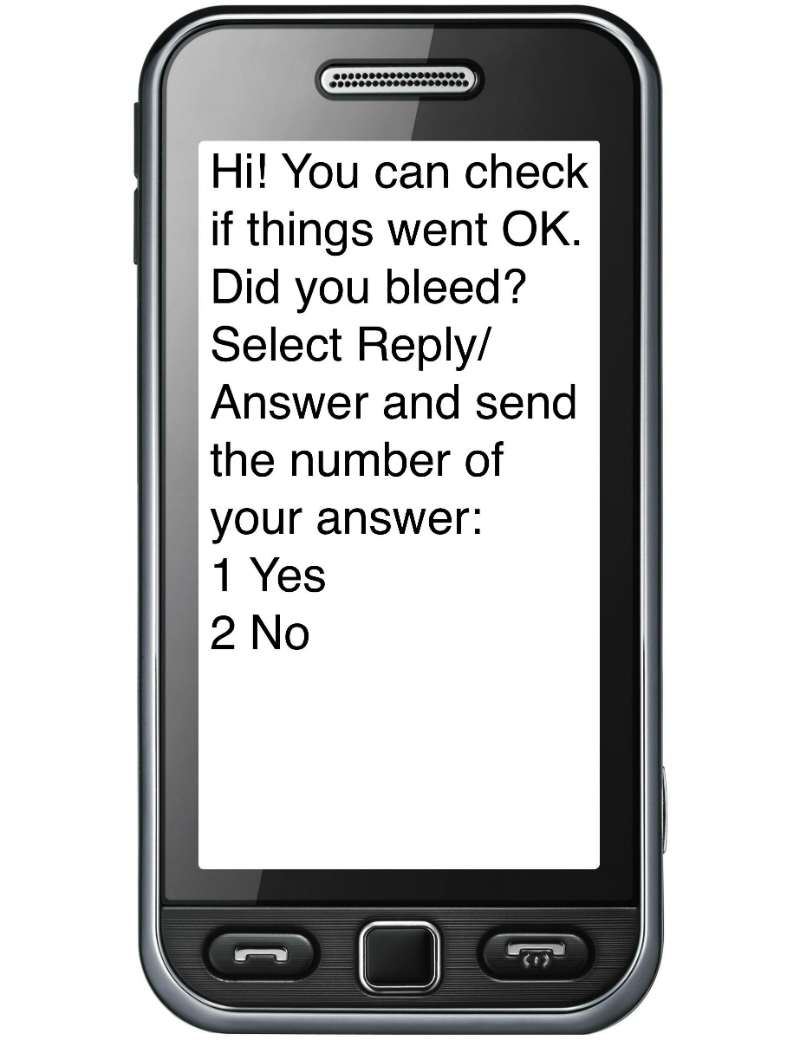
First screen of the self-assessment.

The self-assessment of abortion completion (SA) was a questionnaire designed to be self-administered by participants via mobile 10 days after they had taken the final medical abortion pills, following a text message to complete the questionnaire (Figure 2). Its content was developed based on a literature review and interviews with providers and key informants to understand how abortion completion could be assessed based on questions women would be able to understand and answer. The questionnaire routed users through up to five questions, to assess if the abortion was complete or incomplete, or if pregnancy was ongoing. If the SA concluded that the abortion was incomplete or pregnancy ongoing, the final screen urged users to go back to the clinic for further care.

We decided to provide the SA via mobile phone because a paper-based SA could raise privacy concerns or be lost if clients received it at the clinic on day 1 of the medical abortion process. We also thought that asking participants to do the SA via SMS would be a discreet way of prompting them to do it, and more effective than a pamphlet, which could be lost or easily ignored. There were no privacy concerns with the SA because although its content concerned abortion, it was up to the user to decide when they completed it.

We implemented the SA to be available either via Mxit or via unstructured supplementary service data (USSD) for clients not able or willing to use MXit. USSD is a protocol used by GSM mobile telephones that allows the user to interact with a server via text-based menus. The offering of the SA via USSD meant that an airtime payment had to be made, as USSD costs most users 20 South African cents per 20 seconds. An R10 (approximately $1.10) airtime payment was made on the day they were sent the text message asking them to complete the SA. The SA was implemented in English, Xhosa, and Afrikaans.

#### Family Planning Information

**Figure 3:**
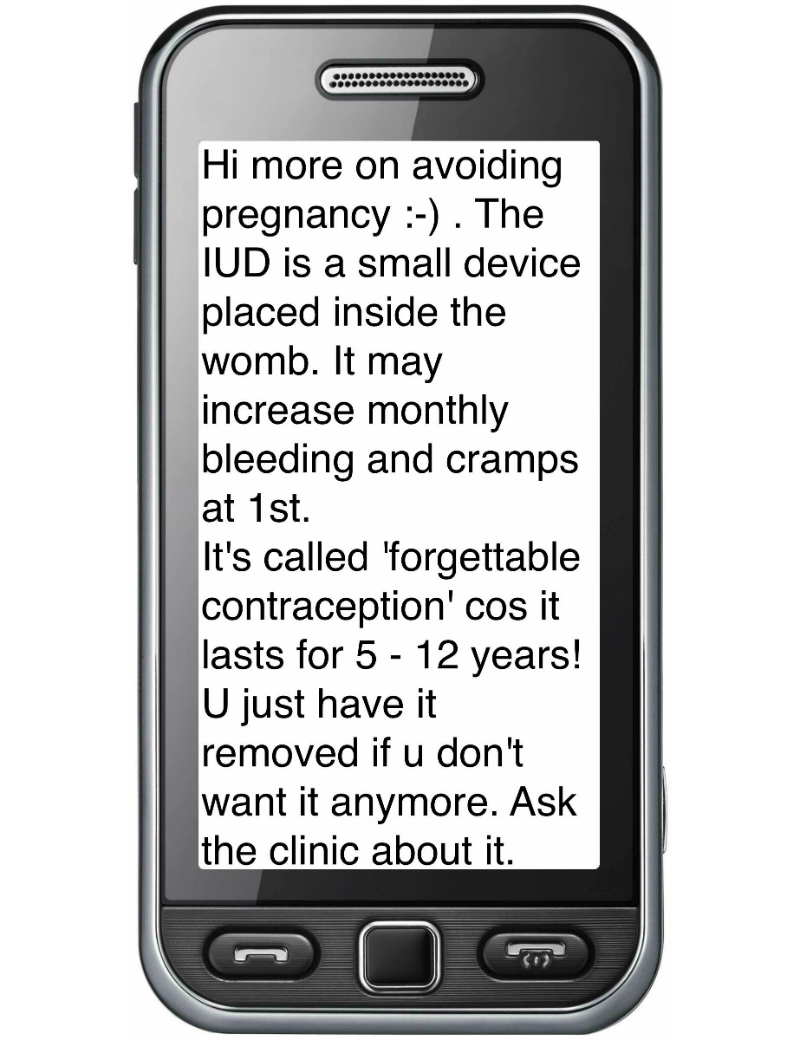
Example of family planning text message.

The family planning content was based on the World Health Organization’s *Family Planning: A Global Handbook for Providers* [18] and study principal investigators’ knowledge of appealing features of various family planning methods (Figure 3). The content was loaded on a mobisite [19], which was also viewable through Mxit. Participants were sent 2 text messages asking them to view the information. The mobisite was created using the free and open source content management system Joomla along with the personal device assistant extension to make the site render well in mobile browsers and Mxit. After the pilot, SMSs providing participants with tips for future choices of family planning methods were added.

### Pilot Study

The pilot of the study was run as a small version of the RCT, and was done to test the mobile interventions and study processes. It did not involve a control arm, and was carried out at the same sites as the NCA from June to September 2011. The pilot was done in two phases due to very low use of the SA in the first pilot and technical problems. Phases 1 and 2 involved 20 and 10 women, respectively.

### Randomized Controlled Trial

This was a two-arm trial in which participants were randomized into a control arm (N=235) and an intervention arm (N=234). Both arms received the standard medical abortion care from the clinics. It was conducted at two nongovernment and two government-run clinics. These were located in Cape Town’s city center; a busy suburban transport terminus; and in Khayelitsha. To be eligible, participants were already signed up to undergo a medical abortion at the clinic, 18 years or older, willing to come to their clinic appointments and be accessible by phone for 7 weeks after consenting, and own a mobile phone on which they could get medical abortion -related information and messaging. A baseline questionnaire was administered after consent (before randomization), and an exit questionnaire was administered at the participant’s follow-up appointment. A telephone interview 1 month after the text messages ended was attempted to probe use of the family planning content provided via mobile phone.

## Results

### Needs and Context Assessment

The NCA provided valuable information on mobile phone use among the study target population, as shown in Table 1.

In terms of mobile phone privacy, 65% (13/20) of clients indicated that no one else used their phone. These other people were most often family members or the client’s boyfriend, while 2 clients said that friends had access to their phone. For text messaging privacy, 80% (16/20) said that no other people ever picked up their phone and read their SMSs. Eighty percent (16/20) indicated that their phone was private enough for them to feel comfortable receiving text messages related to abortion.

In terms of language, 80% of clients (16/20) indicated that they would prefer to receive the text messages in English, while 2 indicated a preference for text messages in Xhosa and 1 preferred Afrikaans. One client would not mind receiving the text messages in Xhosa or English.

Feedback on the text messages indicated that 85% (17/20) thought that they were useful, particularly as a reminder to take their pills; the text messages told them what to expect and provided support. Fifteen percent (3/20) thought that the text messages were not useful because the information was incorrect; the clients didn’t want a reminder because the event was sad; and the client already had the information from the clinic.

**Table 1:** Activities on mobile phone (N=18).^a^

Activity	n (%)
Make/receive calls	18 (100)
Send/receive please-call-me’s	15 (83)
Text messaging	18 (100)
Mxit	3 (17)
Facebook	9 (50)
Email	4 (22)
Browse the Internet	7 (39)

^a^Two missing responses

### Pilot

The pilot showed that the processes and systems used for text messaging initiation and ongoing sending worked well, apart from a Communicate bug experienced in the first pilot that was fixed. The feedback on the text messages showed that most clients were happy with the quantity and content of the messages. All said unequivocally that text messaging helped them through the procedure and that they would recommend them to a friend having a medical abortion. A minority found the messages too long or had difficulties with privacy or problems understanding them.

One participant sent in a please-call-me text message to the designated opt-out number to stop the text messages, on the day that she had received her last text message.

The phase 1 pilot highlighted problems with SA use. Log files provided by the USSD service provider showed that only 65% (13/20) of participants tried the SA. We traced the problem to a bug in Communicate, which meant that the text messages giving participants their airtime on the day that they were to do the SA were not always sent successfully. This was remedied, and in the second pilot, 80% (8/10) of participants tried the SA.

Over both pilots, 70% (21/30) tried the SA and only 48% (10/21) of those who tried were able to complete it successfully. Many users tried the SA multiple times, sometimes with clear patterns of learning (for instance, initially managing to advance one or two screens, and after repeated attempts managing to finish the SA). Based on the pilot results, to maximize SA use, we instituted a short training session on the SA at the time of consent where the fieldworkers went through a simulated SA with the participant on their phones. We also added a manual reminder-text message: if a participant had not completed the SA after 2 text message requests, they were sent a third text message indicating that our logs showed that they had not yet attempted the SA.

Twenty-four clients completed the follow-up telephone interview. Eighty-three percent (20/24) said they had tried to access the family planning information via Mxit or mobisite and of these, 19 (95%) succeeded in reading the information. However, to ensure that everyone in the RCT intervention group received family planning information, we added 5 family planning-related text messages to the campaign of coaching text messages.

### Randomized Controlled Trial

#### Description

This paper does not report on the outcomes of the RCT, which is to be reported elsewhere (paper under submission). Findings relevant to the acceptability and use of mobile phones in the RCT are reported here. In terms of participant numbers, of the 234 randomized to the intervention group at baseline, 8 did not receive the intervention due to invalid numbers, misregistration, system failure, or opt-out, leaving 226 participants receiving the full intervention. Of the 226, 190 (84%) returned and were interviewed at their clinic follow-up visit.

#### Mobile Phone Privacy

At baseline, 86.3% (202/234) said that it was not likely or not possible that someone would see text messages on their phone; 79.5% (186/234) said that others use their phone never or almost never.

However, the exit interviews showed that 20% (38/190) had worried about privacy and receipt of text messages about abortion. Most of those citing privacy concerns were worried about people, particularly family, seeing the text messages:

Because I did not tell anyone at home so I didn't want them to find out before I tell them.

If somebody would have seen the SMSs then they would know what I was doing.

No adverse events relating to mobile phone privacy were reported, however.

#### Text Messaging Language

Text messages were available in English, Afrikaans, and Xhosa in the RCT. No participants chose Afrikaans, and 6.8% (16/234) chose Xhosa.

#### Technical Issues With Text Messaging

A total of 5471 text messages were sent with a failure rate of 5% (that is text messages that could not be delivered due to a network problem or a problem with the user’s phone). Participant comments on the text messages showed that delayed receipt of the text messages was an issue for some, as was repeated deliveries of text messages (the same text message being delivered more than once). Delayed receipt is an inevitable problem because mobile networks can be congested, and users’ phones are sometimes switched off or out of network range. We do not know the reason for repeated deliveries, but assume that it must be an issue on the network.

Qualitative feedback from participants on whether the text messages had helped them through their abortion showed that overwhelmingly, women experienced the text messages as a guide:

It’s almost like they are so informative, they came exactly when you need them and some of the things that SMS told me I was not told at the clinic—so it is like you have a nurse by you all the time.

The SMSs were telling me what to expect like the one that told me that I might vomit and that did happen, but I was not worried because the SMSs had warned me.

I felt that there was someone who knew and understood. They told me what was going to happen.

I didn't have support as I was the only one who knew so the SMSs were there to support and to guide me through the process.

Many also said that they felt supported and comforted, and less alone:

Felt someone was holding my hand through the whole process.

Sometimes the SMSs comforted meI felt the SMSs understood what I was going throughFelt like a friend.

It is a very good thing to have if it was not because of the SMSs, I would have freaked out and come to the clinic—it was like somebody was there looking out for me.

For those who said that some of the text messages made them unhappy, one said that the text messages brought back bad memories, and another said that she was scared to know what was going to happen. Two said that the text messages that talked about blood made them unhappy:

I was expecting that somebody will tell me that I am not going to bleed too much but the SMS I received about bleeding disappointed me.

The SMSs about bleeding and clots is the only one that made me unhappy. I was so scared to see blood.

Comments on the text messages show that some participants shared the text messages. Because this was not an issue that we probed, we do not have statistics on the extent of sharing:

Really opened my eyes. Took me through process. Boyfriend also read SMSs. It made me feel at ease.

They helped me because if I forgot something I will go to my SMSs to remind me. My mother even said: “These people really care about abortion, they even send you SMSs”.

For those who indicated that they did not feel comfortable with the timing of the text messages, comments indicate that daytime might not be the best time to send the text messages (due to the greater likelihood of others seeing the text messages during the day).

Only 1 participant opted out of getting the text messages, on the same day as she had consented to participate in the study. She was called to find out her reasons for opting out, and said that it was for personal reasons.

#### Participant Feedback on the Text Messages

Table 2 summarizes participant feedback on the text messages, which was sought in the exit interview.

**Table 2:** Participant feedback on text messages (N=190).

Question	Answer	n (%)
The text messages were too many	Agree	20 (10.5)
Some text messages were too long	Agree	14 (7.4)
I felt comfortable with the time the text messages were sent to me	Agree	179 (94.2)
The information in the text messages is clear and easy to understand	Agree	99 (52.1)
Some of the text messages made me unhappy	Agree	5 (2.6)
Some of the text messages confused me	Agree	9 (4.7)
I would recommend text messaging to a friend who was going through the same thing	Agree	188 (98.9)
I was always worried about privacy (that someone might see the text messages)	Agree	38 (20)
The text messages helped me through my abortion	Agree	186 (97.9)

#### Self-Assessment

In the exit interview, participants were asked about their use of the SA (Table 3) and most indicated that they had attempted to complete it. Reasons for not completing the SA included not understanding the instructions in the text message, someone else having the phone, and not feeling like it or forgetting. Of those who said they had attempted the SA, most said that the SA was easy or very easy to do. For those who found it hard, difficulties centered on network or system failures. A specific question was asked about failures experienced when attempting the SA, and almost one-fourth of participants reported failures. Participants were asked whether they reached the end of the questions (where it said that the questions were finished), and the vast majority thought that they had.

Computer-generated logs of actual SA use were also analyzed and are shown in Table 4.

Table 5 shows the number of text messages that had to be sent to try to get participants to attempt the SA.

**Table 3:** Self-reported data on self-assessment use.

Outcome	N	n (%)
Attempted the SA	190	175 (92.1)
Reached an endpoint	175	162 (92.6)
SA easy to do	173	161 (93.1)
Failures experienced	177	42 (23.7)

**Table 4:** Computer log data on self-assessment use.

Outcome	N	n (%)
Attempted the SA	226	204 (90.3)
Reached an endpoint	204	176 (86.3; 77.9% of 226)
Reached different endpoints on successive attempts	176	12 (6.9)
Number of attempts needed to reach endpoint	176	1.7 (1.6); 1 (1-2)^a^

^a^Data presented as mean (SD); median (IQR).

**Table 5:** Prompting needed for SA use.

Outcome	N	n (%)
Attempted SA before first text message	226	4 (1.8)
Attempted SA after first text message	226	150 (66.4)
Attempted SA only after second text message	226	29 (12.8)
Were reminded to do SA: third text message	226	38 (16.8)^a^
Responded to third text message and attempted SA	38	21 (55.3)

^a^Five participants were not sent reminders but should have been. Hence, 43/226 (19%) needed a third reminder.

#### Family Planning Information

Of the 234 clients in the intervention arm, 75.6% (177/234) completed telephone interviews 1 month after the text messages ended, although these yielded little useful information on women’s accessing of family planning information via mobile phones. Fifty-two percent (92/177) of those contacted indicated that they had been able to access the family planning information via the mobisite or Mxit (67 did not; 18 had missing data). However, we doubt this self-reported figure because at baseline only 12.8% (30/234) indicated that they used Mxit (we did not ask about mobisite use). Participant comments reinforced what we knew about many of the women not having phones that could access the Internet or Mxit, and reference was made to preferring to access the information via text messaging. Use statistics for the period of the RCT (October 2011 to May 2012) indicate that 813,375 pages of the Mxit/mobisite were viewed, but we cannot match these figures with study participants because site statistics are anonymous.

## Discussion

### Principal Results

This paper focused specifically on findings related to the development and use of the mobile phone-based interventions provided in the study. Our main findings were that the extensive formative research process led to the development of text messages that were highly acceptable and a valued form of coaching through medical abortion; a mobile phone-based questionnaire to check abortion progress was viable in terms of use, given a short training session; and phone privacy in the study context was not perceived to be a major issue, although in practice more women worried about privacy than had indicated this at baseline.

While a three-stage study design added to study time and cost, it was essential to assess how people in our target population used their phones so that we could tailor what mobile technologies (eg, Mxit, USSD, and SMS) were appropriate for delivering the interventions. The pilot phase allowed us to do usability testing, particularly for the SA, and showed us the importance of a short training on how to do it.

It is of particular interest that the text messages were seen as supportive, like someone was there for the recipient. They were not written to be overtly supportive; the text messages did not deal with the decision to terminate, or say things that specifically indicated support. Rather, they focused on the practical aspects of medical abortion, and sought to reassure about what physical experiences are normal. Feeling supported could have arisen from the content and scheduling of the text messages matching the women’s experiences and so they felt understood. The feature of text messages being a push technology was important for scheduling: Push means that the recipient does not have to do anything to receive the messages; they arrive on the phone without the recipient having to take an action other than consenting to receive the text messages. The push nature of text messages meant that we could time receipt of information according to the expected process of a medical abortion and deliver the messages in a medium that made it more likely that they would be read on or close to receipt.

Gurman [15] has indicated that timing of text messages is an area needing research; this study has indicated that morning and early evening were acceptable to the large majority (text messages were sent around 9 am and 6 pm). Given that a few participants raised timing-related privacy concerns (eg, receipt of text messages at work), it would be ideal to allow recipients to choose the time of receipt of text messages.

In terms of language choice for the text messages, a strong preference for English was indicated in the NCA. This was borne out in the RCT, even though 2 of the 4 study sites were in a predominantly Xhosa-speaking area. This preference among non-first language speakers for English texts is true of many of Cell-Life’s other (unpublished) instances of text messaging use in health, although it is not always the case.

While most participants did not identify phone privacy as an issue, the RCT showed that having received the text messages, more participants had privacy concerns than had indicated this at signup. This is not surprising because receipt of text messages during abortion would have been unfamiliar to the participants. We suggest that similar interventions should consider two ways of mitigating privacy concerns. The first is showing potential recipients examples of text messages they will receive that may lead to privacy problems, to help them decide if they are comfortable receiving such text messages. Second, a way of allowing recipients to stop the text messages should be provided and recipients should be periodically reminded of this during the time that they receive the text messages.

There was high, successful use of the SA questionnaire, and most women completed their self-assessment in good time for them to take action on the result, if they needed to. This shows promise for other medical procedures where self-assessment via mobile by patients could relieve burden on them or health care providers. Women may have been motivated to complete the SA because they were aware that they were part of a study, although the fact that they followed standard-of-care (which includes a follow-up appointment to assess abortion completion) may have demotivated some of them. The airtime sent to women before they were asked to complete the SA should not have acted as an incentive to do it, because participants received the airtime whether they attempted the SA or not.

The fact that many more women thought they had finished the SA than actually had indicates that such a system should include a mechanism to feed back to the user whether they have completed the assessment.

### Comparison With Prior Work

This study’s findings on using text messages to drive SA use is in keeping with other studies that have shown that SMSs can be an effective spur to action in a health care context, such as getting women to perform breast self-examination [20] or take an HIV test [21].

### Potential Reuse of the Mobile Interventions

The text messages used in this study can be used by medical abortion providers in other locations, although a localization process would be recommended (testing of text messages, and adaptation of content for local conditions). The SA questionnaire could be used in different settings, although the questionnaire was not satisfactory in detecting ongoing pregnancy (these results will be reported elsewhere). The family planning information is available at [19]. The study protocol, instruments, SMS text messages, presentations, and other media are available at [22].

### Conclusions

To explore the use of mobile phones to simplify medical abortion provision, a study was done in South Africa to assess the feasibility and efficacy of text messages to coach women through medical abortion, a questionnaire assessing medical abortion completion via mobile phone, and family planning information provided via mobile. This paper described the process of developing these mobile interventions, and showed the importance of assessing the context and piloting. It reported on findings of the study related to mobile phone use in this context. Text messages provided a good medium for timed, push information that guided and supported women through medical abortion. Women were able to complete a self-assessment questionnaire via mobile phone if given a short training session. Phone privacy was not as much of an issue as we thought it might be, although it concerned more women after they had received the intervention, than indicated that it would be an issue at baseline. It is hoped that this study may contribute to the successful expansion of medical abortion provision aided by a technology that is increasingly ubiquitous—mobile phones.

## Abbreviations

AMPS: All Media and Products Survey
ITU: International Telecommunications Union
NCA: needs and context assessment
RCT: randomized controlled trial
SA: self-assessment of abortion completion
SMS: short message service
USSD: unstructured supplementary service data
